# Zinc oxide nanoparticles improve lactation and metabolism in dairy goats by modulating the rumen microbiota

**DOI:** 10.3389/fmicb.2024.1483680

**Published:** 2024-11-25

**Authors:** Shan Xie, Zaixiang Ying, Ziqing Xiu, Yawang Sun, Qinlin Yang, Hanyu Gao, Wenqiao Fan, Yongjiang Wu

**Affiliations:** ^1^College of Smart Agriculture, Chongqing University of Arts and Sciences, Yongchuan, China; ^2^College of Biology and Food Engineering, Chongqing Three Gorges University, Wanzhou, China; ^3^College of Animal Science and Technology, Southwest University, Beibei, China; ^4^Chongqing Institute of Medicinal Plant Cultivation, Nanchuan, China

**Keywords:** zinc oxide nanoparticles, dairy goats, lactation, rumen microbiota, metabolomics

## Abstract

This study aimed to investigate the effects of dietary supplementation with zinc oxide nanoparticles (ZnONPs) on lactation, rumen microbiota, and metabolomics in dairy goats. Twenty Guanzhong dairy goats, with comparable milk yields and in the mid-lactation stage, were randomly divided into two groups, with 10 goats in each group. The control group was fed a standard diet, while the ZnONP group received the control diet plus 30 mg ZnONPs/kg DM. The pre-trial period lasted for 7 days, followed by a trial period of 30 days. The results showed that the addition of ZnONPs increased the milk yield and milk fat content (*p* < 0.05). The results of rumen microbial sequencing showed that the Chao1, Observed species, and PD_whole_tree indices of the ZnONP group were higher than those of the control group. The addition of ZnONPs altered the composition of the rumen microbiota, increasing the abundance of beneficial bacteria (*Prevotella* and *Rikenellaceae_RC9_gut_group*) and decreasing the abundance of the harmful bacterium *Sediminispirochaeta*. Non-targeted metabolomics analysis identified a total of 261 differential metabolites between the two groups, indicating changes in rumen metabolism. Further correlation analysis revealed a positive correlation between beneficial bacteria (*Rikenellaceae RC9 gut group* and *Anaeroplasma*) and metabolites such as nicotinamide riboside, inosine, and guanosine (*p* < 0.05). In addition, a positive correlation was observed between milk yield and beneficial bacteria (*RF39* and *Clostridia vadinBB60 group*), as well as between milk fat content and *Quinella* (*p* < 0.05). In summary, ZnONP supplementation can improve the structure of the rumen microbiota in dairy goats, positively influencing milk yield, milk composition, and metabolism.

## 1 Introduction

In modern dairy production, the milk yield and metabolic health of dairy goats are crucial for improving farming efficiency. Zinc, as an essential trace element, is a necessary component of various proteins in animal bodies (Swain et al., 2016). It plays a significant role in enhancing production performance, regulating metabolism, promoting the digestion and absorption of nutrients, and maintaining intestinal health (Kujur et al., 2016). In animal diets, the primary issue with using inorganic zinc as a feed supplement is its relatively low bioavailability. To achieve optimal production performance, the dietary zinc content used in animal husbandry often exceeds the recommended safe supplementation levels (Bratz et al., 2013). However, excessive use can lead to zinc overload and environmental pollution (Komatsu et al., 2020).

In recent years, the application of nanotechnology in agriculture has garnered significant attention as it effectively enhances the bioavailability of trace elements in animal diets while simultaneously reducing the risk of environmental pollution (Gopi et al., 2017). In agricultural production, the application of low doses of zinc oxide nanoparticles (ZnONPs) has been shown to have no negative impact on the health and stability of ecosystems, making it a safe and efficient zinc fertilizer (Sun et al., 2022; Xu et al., 2023). Compared to traditional zinc sources, ZnONPs possess advantages such as smaller particle size, larger specific surface area, and higher chemical reactivity, making it a feed additive with potential application value (Mandal et al., 2022). ZnONPs can act as molecular channels and carriers within cells, enhancing the transport and transmission of nutrients and signaling molecules, thereby providing the essential material basis for normal cell growth (Mokone et al., 2022). Compared to conventional zinc oxide, ZnONPs can enhance zinc bioavailability and increase serum carbonic anhydrase activity and growth hormone levels (Tsai et al., 2016). Dietary supplementation with ZnONPs (20 mg/kg) effectively improved the growth performance and feed conversion ratio of broilers, compared to the conventional dose of 60 mg/kg of zinc oxide (Zhao et al., 2014). Similarly, ZnONPs (30 mg/kg) as a replacement for conventional zinc sources have been shown to effectively improve the growth performance and meat quality of New Zealand White rabbits, under heat stress conditions (Kamel et al., 2020; Abdel-Wareth et al., 2022). This is because zinc in nanoparticle form can quickly penetrate the gastrointestinal membrane into the bloodstream, promoting the digestion and absorption of nutrients in the feed and thereby enhancing production performance (Liao et al., 2010). In addition, the bioavailability of zinc in the body plays a crucial role in maintaining and enhancing immune responses (Yusof et al., 2019). Dietary supplementation with ZnONPs (80 mg/kg) increased the antibody titer in laying hens compared to conventional zinc oxide, indicating a better immune response (Abedini et al., 2018). In summary, ZnONPs are more effective than traditional zinc sources and can be used as substitutes for conventional zinc sources, thereby reducing the effective dosage of zinc.

The richness and diversity of intestinal microbiota have a significant impact on maintaining intestinal homeostasis and nutrient absorption (Kim and Isaacson, 2015; Mukherjee et al., 2020). ZnONPs can regulate the balance of intestinal microbiota in weaned piglets, promoting the proliferation of beneficial bacteria and inhibiting the growth of harmful bacteria, thereby reducing the invasion of gastrointestinal pathogens (Xia et al., 2017; Pei et al., 2019). Research indicates that altering cell functions can affect the body’s metabolism, and ZnONPs can effectively change the levels of metabolites such as glucose and amino acids in broiler chicken plasma, especially *β*-glucose (Feng et al., 2017). Pearce et al. (2015) found that dietary organic zinc can improve the production of markers associated with muscle breakdown, such as reducing plasma urea nitrogen, while increasing lysozyme levels, thereby alleviating the adverse effects of heat stress on pigs. However, excessive ZnONPs can disrupt energy metabolism in mice’s kidneys, causing damage to mitochondria and cell membranes (Yan et al., 2012). Therefore, the concentration of ZnONPs in animal diets should be controlled at a specific minimum level to prevent its potential toxic effects.

Existing applications of ZnONPs are primarily focused on animal production in piglets and poultry, with fewer research findings related to ruminant production. Therefore, this experiment aims to investigate the effects of dietary supplementation with ZnONPs on the lactation, rumen microbiota, and metabolomics of dairy goats. The purpose is to provide guidelines for the application of ZnONPs in ruminant production.

## 2 Materials and methods

### 2.1 Experimental material

ZnONPs were provided by Shanghai Macklin Biochemical Co., Ltd., with a purity of 99.9%. The average particle size is 30 ± 10 nm, and it is uniformly dispersed without agglomeration. The CAS number is 1314-13-2.

### 2.2 Ethical considerations

The research protocol for this project has been reviewed by the Ethics Committee of Chongqing University of Arts and Sciences (approval number, CQWLDF0029).

### 2.3 Experimental design

The experiment was conducted at the Black Goat Breeding Base in Chongqing, China. Twenty Guanzhong dairy goats, with comparable milk yields and in the mid-lactation stage, were randomly divided into two groups, with 10 goats in each group. The control group was fed a standard diet, while the ZnONP group received the control diet plus 30 mg ZnONPs/kg DM. The composition and nutritional levels of the standard feed are shown in Table 1. The control group and the ZnONP group were housed individually, with identical temperature, humidity, and management conditions. The feeding times were set at 7 a.m. and 7 p.m. daily. One week prior to the experiment, a thorough check of ventilation and cooling equipment was conducted to ensure they were functioning properly, and the necessary sheep pens were thoroughly cleaned and disinfected. The pre-trial period lasted 7 days, followed by a 30-day trial period.

**Table 1:** Composition and nutrient levels of the basal diet (DM basis) %.

Items	Content
Ingredients	
Alfalfa hay	40.00
Corn	35.76
Soybean meal	11.41
DDGS	6.25
Corn germ meal	3.58
NaCl	0.74
CaHPO_4_	0.61
Limestone	0.65
Premix^a^	1.00
Total	100.00
Nutrient levels	
Dry matter	87.12
Metabolic energy/(MJ/kg)^b^	10.46
Crude protein	12.25
Neutral detergent fiber	31.03
Acid detergent fiber	17.14
Ca	0.66
Zn	0.004
Phosphorus	0.2

a The premix provided the following per kg of the diet: VA 7000 IU, VD 1800 IU, VE 40 IU, Cu 12 mg, Fe 60 mg, Mn 50 mg, Zn 40 mg, I 1.0 mg, Se 0.27 mg, Co 0.3 mg.

b Metabolic energy is the calculated value, while the rest are measured values.

### 2.4 Collection and processing of experimental samples

Before each feeding, the remaining feed was removed from the feed trough and weighed using an electronic scale. The residual amounts of both concentrate and roughage were recorded daily. At the end of the experiment, these data were used to calculate the dry matter intake (DMI) of the two groups of dairy goats. An automatic milking machine with vacuum suction was used for milking, and the daily milk yield of each dairy goat was recorded. The milk quality was measured every 2 days using the following sampling method: Manual milking was performed on two groups of dairy goats at 08:00 in the morning and 20:00 in the evening. The collected milk samples were placed in labeled 50 mL centrifuge tubes. The morning and evening milk samples were mixed at a ratio of 3:2 and stored at −20°C for the determination of milk quality parameters.

At 30d of the trial period, rumen fluid was collected from two groups of dairy goats using an oral stomach tube, filtered through four layers of sterile gauze, and aliquoted into 2 mL cryovials. The cryovials were wrapped with sealing film, flash-frozen in liquid nitrogen, and stored at −80°C. These samples were used for subsequent rumen microbiome 16S rDNA sequencing and non-targeted metabolomics analysis.

### 2.5 Compositional analysis

The collected milk samples from the two groups of dairy goats were heated to 37°C using a water bath. The contents of fat, protein, lactose, non-fat milk solids, and total solids were measured using a LACTOSCAN MCCW milk analyzer.

### 2.6 Rumen microbiome 16S rDNA analysis

The total DNA of rumen fluid bacteria was extracted using the E.Z.N.A. Soil DNA Kit. The DNA quality and concentration were measured with a UV spectrophotometer (NanoDrop 2000). The V3–V4 regions of the bacterial 16S rDNA gene were amplified using universal primers 338F (5′-ACTCCTACGGGA-GGCAGCAG-3′) and 806R (5′-GGACTACHVGGGTWTCTAAT-3′). An 8 bp barcode sequence was added to the 5′ ends of the upstream and downstream primers to distinguish different samples. The universal primers with barcode sequences were synthesized, and the amplification was carried out on an ABI 9700 PCR instrument (Applied Biosystems, Inc., United States). After purifying the PCR amplification products, the concentration was measured, and high-throughput sequencing was performed on the Illumina NovaSeq sequencing platform. The sequencing was conducted at Beijing Allwegene Technology Co., Ltd.

The sequencing data were processed using Pear software (Zhang et al., 2014) (v0.9.6) to filter, merge, and remove chimeras, resulting in high-quality sequences. Subsequently, high-quality sequences were clustered into operational taxonomic units (OTUs) using the UPARSE (Edgar, 2013) algorithm in Vsearch software (Rognes et al., 2016) (v2.7.1) with a sequence similarity threshold of 97%. The representative OTU sequences were then compared against the Silva138 (Pruesse et al., 2007) database using the BLAST (Ye et al., 2006) algorithm, with an *e*-value threshold set at 1e-5, to obtain the taxonomic classification information of the OTUs. Based on OTU and abundance results, *α*-diversity and *β*-diversity indices were calculated using QIIME (v1.8.0) software and visualized using R (v3.6.0). In conjunction with species annotation and relative abundance data, species composition bar chart analysis was conducted using R (v3.6.0). In addition, LEfSe (Segata et al., 2011) analysis was performed using Python (v2.7) to identify significantly different biomarkers. The functional composition of the microbiome was predicted using PICRUSt2 software.

### 2.7 Rumen metabolomics analysis

Rumen fluid samples were removed from a −80°C freezer and thawed. Then, 100 μL of the sample was transferred to an EP tube, and 400 μL of extraction solution (methanol: acetonitrile = 1:1 (V/V), containing an isotopically labeled internal standard mixture), was added. The mixture was vortexed for 30 s and then ultrasonicated for 10 min in an ice-water bath. The sample was then left to stand at −40°C for 1 h. Subsequently, the sample was centrifuged at 4°C, 12,000 rpm (13,800 × g relative centrifugal force, radius 8.6 cm) for 15 min. The supernatant was transferred into an injection vial for analysis. In addition, equal amounts of supernatant from all samples were combined to create a QC sample for machine testing. Metabolite separation and detection were performed using a Thermo Fisher Vanquish UHPLC system coupled with a Thermo Fisher Q Exactive™ HF-X mass spectrometer.

Metabolite information was annotated using the HMDB, METLIN, and KEGG databases, followed by partial least squares discriminant analysis (PLS-DA) performed using R software. The metabolites with VIP > 1 and *p* < 0.05 (Student’s *t*-test) and Fold Change < 0.67 or Fold Change > 1.5 were considered as significantly changed metabolites. Furthermore, the annotated differential metabolites were mapped to the KEGG pathway database using the KEGG database.^1^

### 2.8 Statistical analysis

Data on production performance, dry matter intake, and milk composition were analyzed using Student’s *t*-test with SPSS version 27.0. The results are presented as means with standard error of the mean (SEM), and statistical significance was set at a *p-*value < 0.05. The correlation between rumen microbiota, metabolites, and milk composition indices was analyzed using the psych package in the R project for Spearman’s correlation. A *p*-value < 0.05 was considered statistically significant.

## 3 Results

### 3.1 DMI, milk production, and milk composition

Table 2 shows that, compared to the control group, the milk yield in the ZnONP group was higher (*p* < 0.05) with no effect on dry matter intake (DMI). The ZnONP group had a higher milk fat content than the control group (*p* < 0.05), while there were no differences in milk protein, lactose, total solids, and non-fat milk solids. The energy-corrected milk yield (ECM) in the ZnONP group was higher than in the control group (*p* < 0.05).

**Table 2:** Effects of ZnONP supplementation in diet on intake and milk production in dairy goats.

Items	CK	ZnONPs	SEM	*p*-value
DMI, kg/d	20.57	20.65	0.23	0.720
Milk, kg/d	7.63	8.38	0.12	<0.001
ECM, kg/d	5.92	6.91	0.49	0.049
Fat, g/kg	26.19	28.52	0.86	0.012
Protein, g/kg	36.17	35.16	1.10	0.365
Lactose, g/kg	37.77	37.15	1.82	0.733
Total solids, g/kg	6.15	6.05	1.81	0.566
Non-fat milk solids, g/kg	80.22	78.52	1.60	0.298

DMI, dry matter intake; ECM, energy-corrected milk; SEM, standard error of the mean. CK, dairy goats fed a standard diet. ZnONPs, dairy goats fed a standard diet plus 30 mg ZnONPs/kg DM.

### 3.2 Rumen microbiome 16S rDNA sequencing analysis

#### 3.2.1 The effects of ZnONPs on the rumen microbiota community in dairy goats

**Figure 1:**
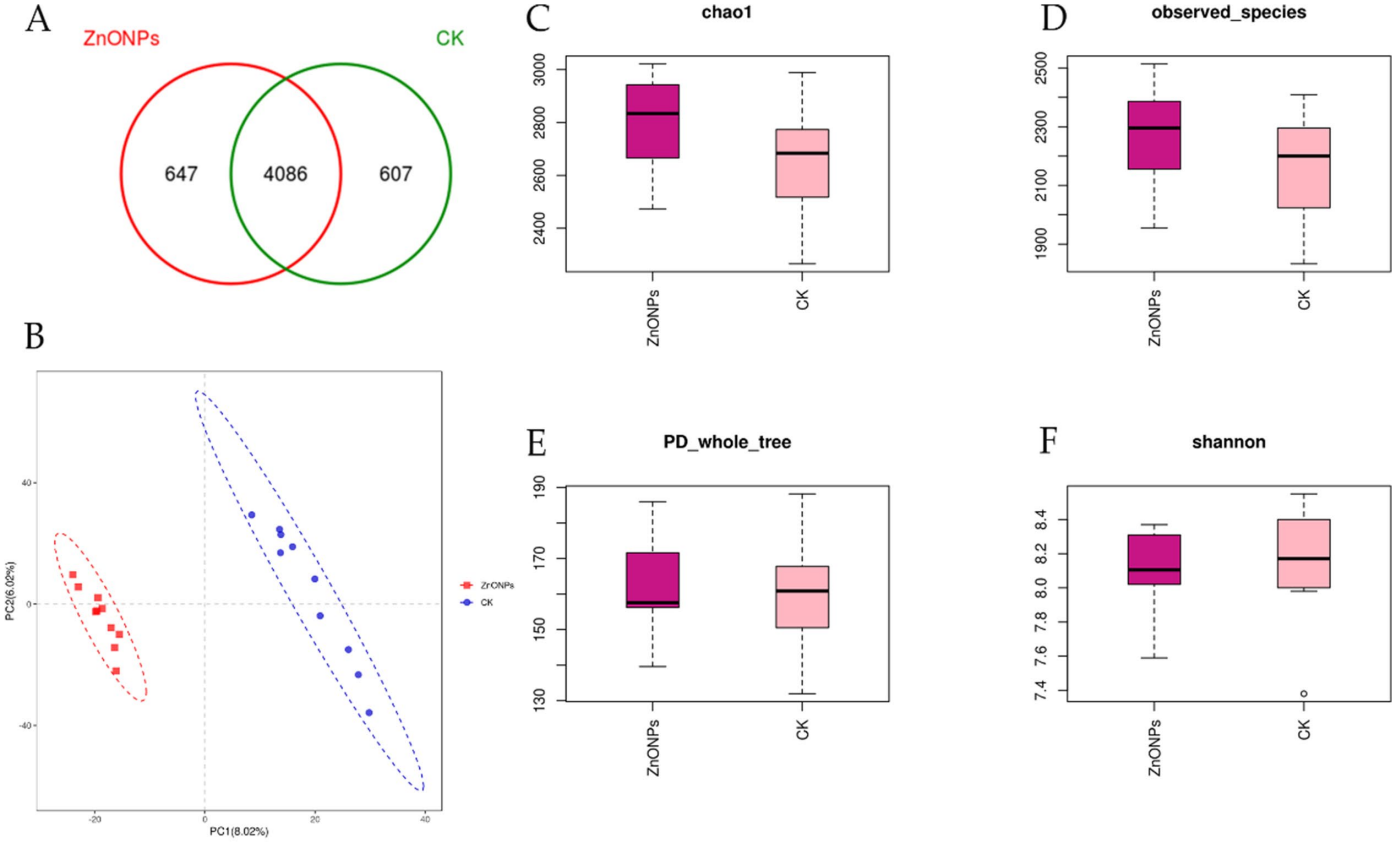
Comparison of rumen microbial diversity between the two groups. The Venn diagram shows the number of shared or unique OTUs **(A)**. PLS-DA analysis based on OTUs **(B)**. Box plot of Chao1 index **(C)**. Box plot of Observed species index **(D)**. Box plot of PD_whole_tree index **(E)**. Box plot of Shannon index **(F)**. PD_whole_tree index, the diversity index based on phylogenetic trees indicates that a higher value corresponds to greater community diversity. CK, dairy goats fed a standard diet. ZnONPs, dairy goats fed a standard diet plus 30 mg ZnONPs/kg DM.

For the sequencing of 20 rumen fluid samples from the two experimental groups, a total of 1,434,622 sequences were obtained. After quality control and subsequent removal of chimeras and short sequences, a total of 1,413,649 sequences were obtained, resulting in the formation of 5,340 OTUs. As shown in Figure 1A, the ZnONP group had 647 unique OTUs, while the control group had 607 unique OTUs, with 4,086 OTUs shared between the ZnONP group and the control group.

Alpha diversity indices for the 20 samples were computed using the QIIME software (Table 3). The Goods coverage reflects the coverage of low-abundance OTUs in the samples, with both groups having a coverage of 0.99, close to 1. This indicates that the sequencing results effectively cover the majority of bacteria in the rumen. Compared to the control group, the ZnONP group exhibited increased Chao1 and Observed species indices, indicating an increase in species richness within the community (Figures 1C,D). Following the addition of ZnONPs to the diet, the PD_whole_tree index of rumen microbiota increased, while the Shannon index decreased, indicating an increase in microbial diversity and a decrease in evenness (Figures 1E,F). By employing partial least squares discriminant analysis (PLS-DA) to investigate the beta diversity of the samples, it was found that samples within the control group and the ZnONP group tended to cluster internally, with a notable separation between groups, indicating a significant difference in the rumen microbiota composition structure between the two groups (Figure 1B).

**Table 3:** Rumen flora richness and diversity index.

Group	Chao1	Observed species	PD_whole_tree	Shannon
CK	2649.29 ± 216.38	5158.49 ± 192.87	159.49 ± 15.03	8.14 ± 0.33
ZnONPs	2806.78 ± 174.02	2267.73 ± 165.10	161.03 ± 14.28	8.07 ± 0.28

Data are presented as mean ± SEM. CK, dairy goats fed a standard diet. ZnONPs, dairy goats fed a standard diet plus 30 mg ZnONPs/kg DM.

#### 3.2.2 The effects of ZnONPs on the relative abundance of rumen microbiota in dairy goats

**Figure 2:**
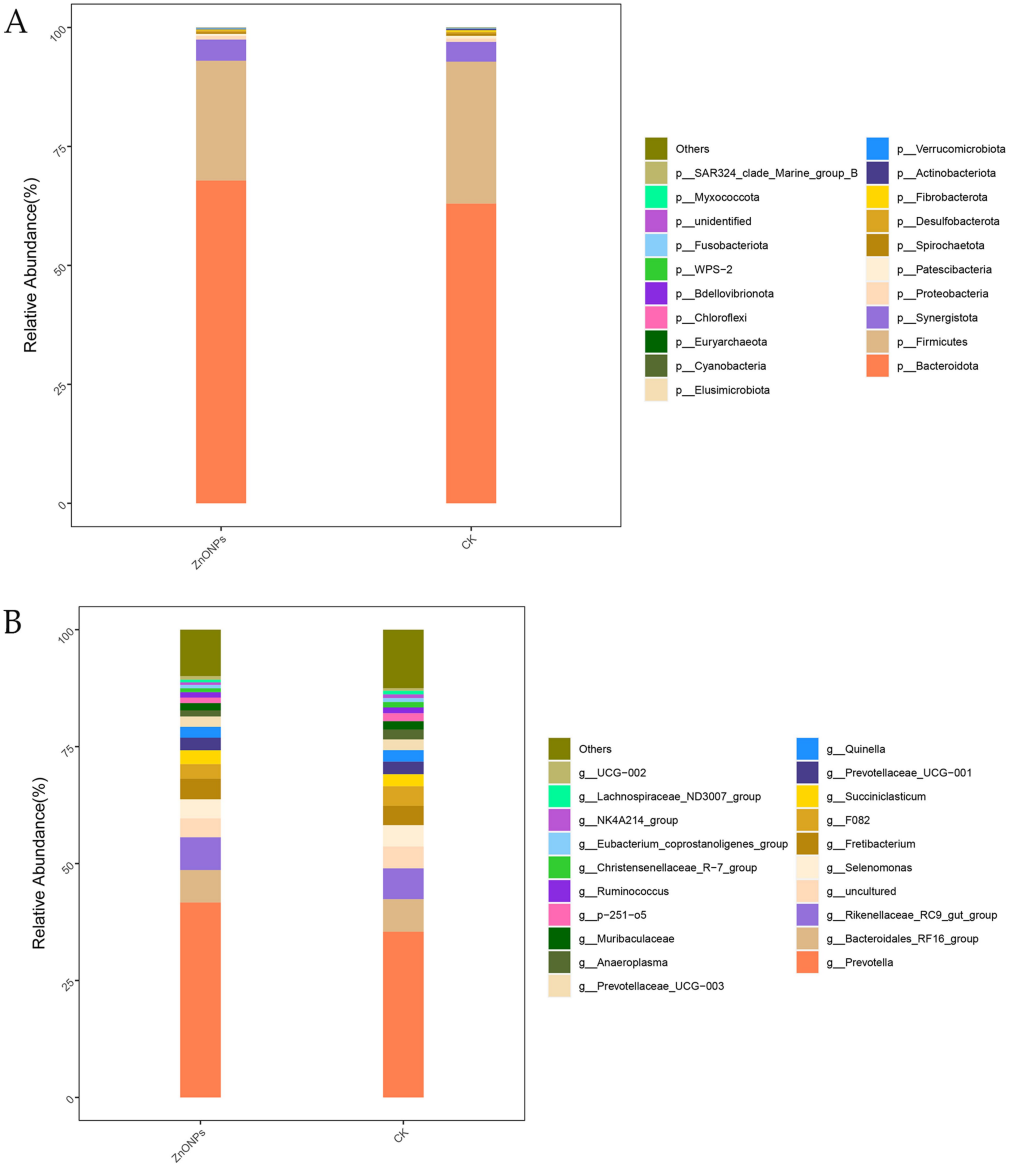
Bar chart of rumen microbiota species composition analysis. The tax level of phylum **(A)** and the tax level of genus **(B)**. CK, dairy goats fed a standard diet. ZnONPs, dairy goats fed a standard diet plus 30 mg ZnONPs/kg DM.

Based on the OTU annotation results, a total of 25 phyla, 41 classes, 78 orders, 122 families, and 232 genera were identified across the two groups. As shown in Figure 2A, at the phylum level, the rumen microbiota of dairy goats is mainly composed of *Bacteroidota*, *Firmicutes*, and *Synergistota*, with *Bacteroidota* being the dominant phylum. Compared to the CK group, after the addition of ZnONPs, the relative abundance of *Bacteroidota* and *Synergistota* increased, while the relative abundance of *Firmicutes* decreased. At the genus level, compared to the CK group, the ZnONP group showed an increase in the relative abundance of *Prevotella* and *Rikenellaceae_RC9_gut_group*, while the relative abundance of *Selenomonas* decreased (Figure 2B). The relative abundance levels of rumen microbiota are shown in Supplementary Tables S1, S2.

#### 3.2.3 Rumen microbiota differential species analysis

**Figure 3:**
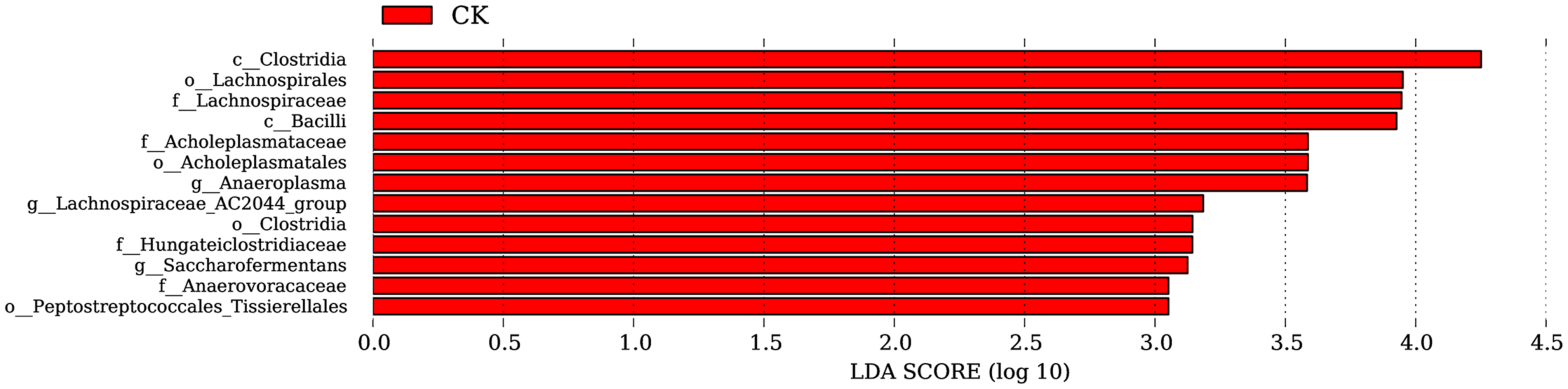
Significantly different bacterial taxa identified by the linear discriminant analysis effect size. Red represents CK. CK, dairy goats fed a standard diet.

Based on the annotation information, LEfSe analysis revealed no significantly dominant species in the ZnONP group. In the control group, a total of 13 significantly different populations were identified within the rumen microbiota (all with LDA values greater than 2) (Figure 3). Among these populations, *Clostridia, Lachnospirales, Lachnospiraceae, Bacilli, Acholeplasmataceae, Acholeplasmatales*, and *Anaeroplasma* were predominant. The class *Clostridia* are the primary microorganisms in the *Firmicutes* phylum responsible for the metabolism of carbohydrate substances. The orders *Lachnospirales* and *Lachnospiraceae* are considered potentially beneficial bacteria involved in the metabolism of various carbohydrates.

#### 3.2.4 Prediction of rumen microbial functions

**Figure 4:**
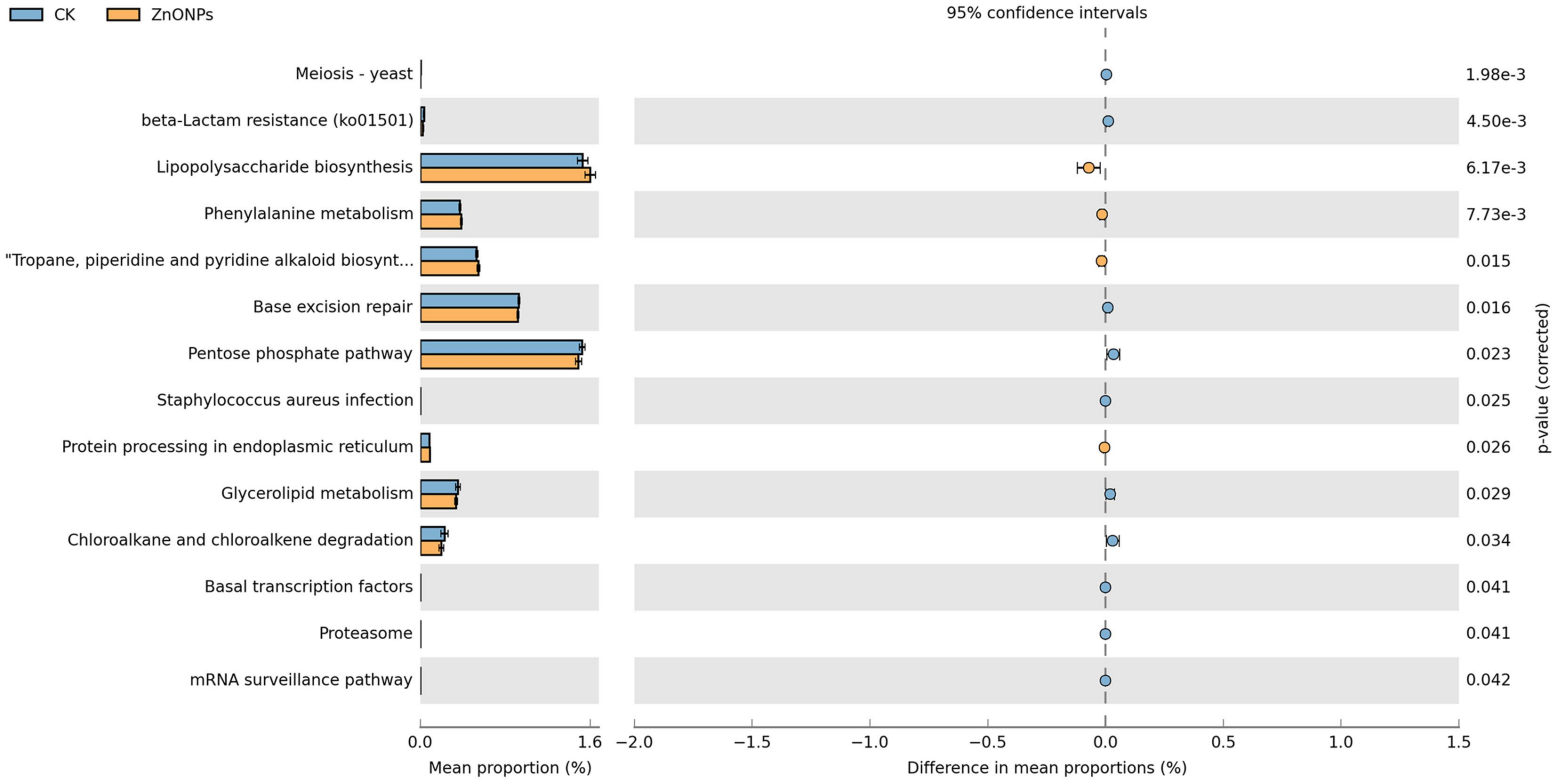
Rumen microbial functional analysis. Blue represents CK, and yellow represents ZnONPs. CK, dairy goats fed a standard diet. ZnONPs, dairy goats fed a standard diet plus 30 mg ZnONPs/kg DM.

As shown in Figure 4, the functional activities of rumen microbiota are primarily focused on lipopolysaccharide biosynthesis, pentose phosphate pathway, base excision repair, and tropane, piperidine, and pyridine alkaloid biosynthesis. Through Welch’s *t*-test for differential analysis, a total of 14 pathways were screened out with differential genes between the two groups. Comparative analysis between the control group and the ZnONP group revealed that the rumen microbiota community in the ZnONP group exhibited enhancements in pathways such as lipopolysaccharide biosynthesis, phenylalanine metabolism, and tropane, piperidine, and pyridine alkaloid biosynthesis (*p* < 0.05), while pathways including the pentose phosphate pathway, chloroalkane and chloroalkene degradation, and glycerolipid metabolism showed attenuations (*p* < 0.05). The functional genes of rumen microbiota were primarily classified under carbohydrate metabolism functions.

### 3.3 Rumen microbial non-targeted metabolomics analysis

#### 3.3.1 Rumen metabolite multivariate statistical analysis

**Figure 5:**
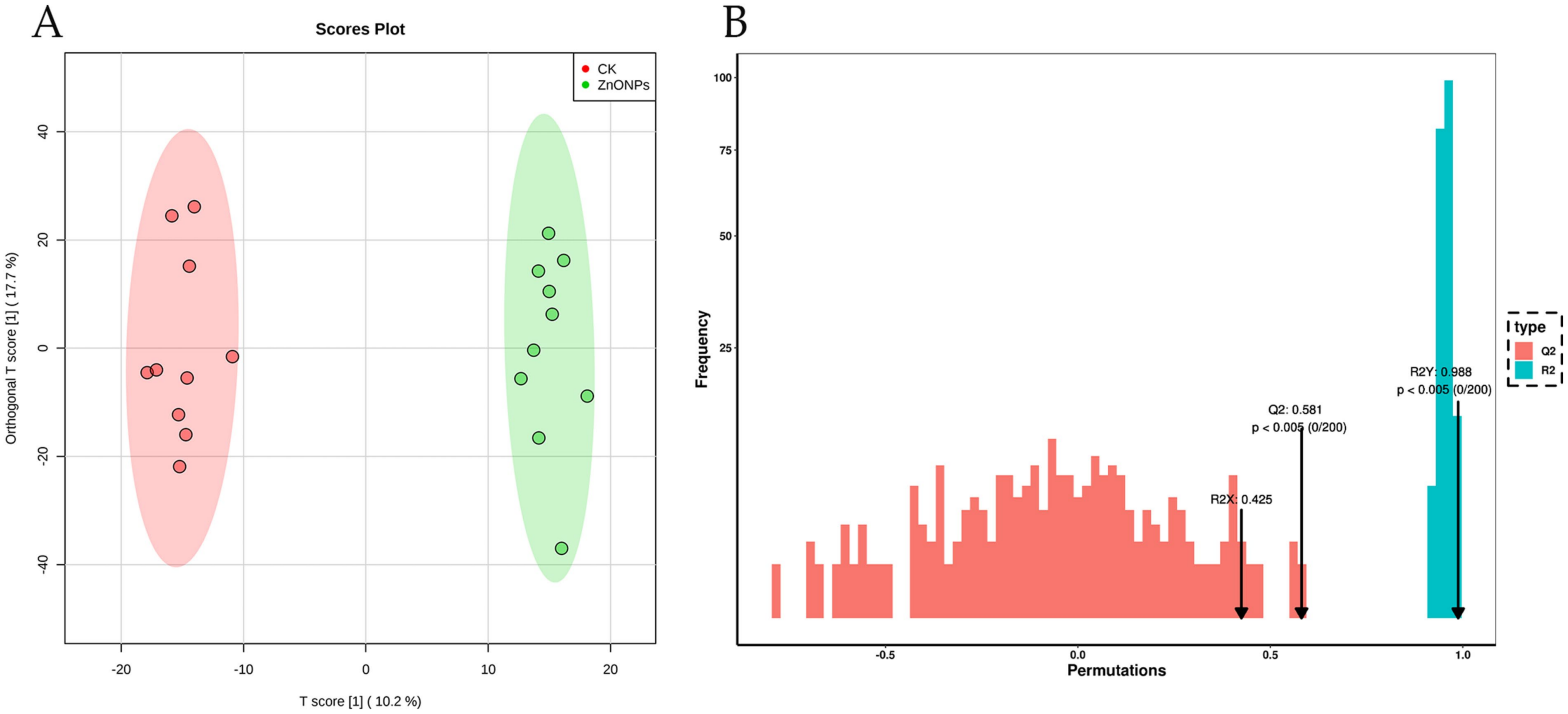
OPLS-DA score plot for the CK and ZnONP groups **(A)**. Red represents CK, and green represents ZnONPs. OPLS-DA model validation plot **(B)**. CK, dairy goats fed a standard diet. ZnONPs, dairy goats fed a standard diet plus 30 mg ZnONPs/kg DM.

As shown in Figure 5A, there is a clear separation of metabolites between the two experimental groups, with a well-defined clustering pattern, indicating significant differences between the metabolites of the ZnONP group and those of the control group. To assess the presence of overfitting in the OPLS-DA model, permutation tests were conducted for statistical validation of the OPLS-DA model. R2Y and Q2 represent the explanatory and predictive abilities within the OPLS-DA model, respectively, with values closer to 1 indicating greater stability and reliability of the model. A Q2 value greater than 0.5 signifies a good predictive capacity of the model. With R2Y = 0.988 and Q2 = 0.581, the model demonstrates good predictive ability and reliable results (Figure 5B).

#### 3.3.2 The effects of ZnONPs on the rumen metabolites of dairy goats

**Figure 6:**
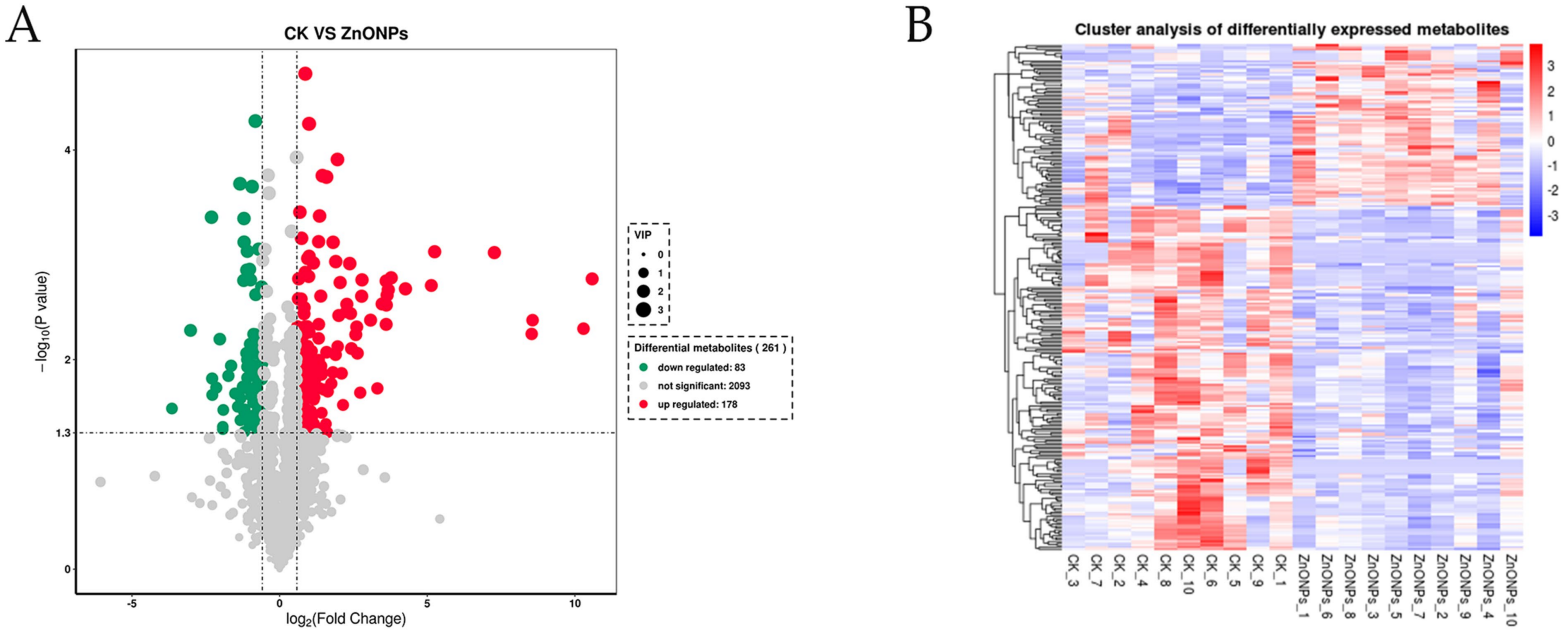
Volcano plot of intergroup differential metabolites **(A)**. Red represents significantly upregulated metabolites, green represents significantly downregulated metabolites, and gray represents non-significantly changed metabolites. The size of the dots represents the VIP value. Heatmap of clustering analysis of rumen differential metabolites **(B)**. The color blocks at different positions represent the relative expression levels of the corresponding metabolites at those locations. CK, dairy goats fed a standard diet. ZnONPs, dairy goats fed a standard diet plus 30 mg ZnONPs/kg DM.

Through a combination of univariate statistical analysis and multivariate statistical analysis methods, we screened for differential metabolites between the groups. The screening criteria used were a *p*-value < 0.05, VIP ≥ 1, and Fold Change < 0.67 or > 1.5. The results of the screening were visualized in a volcano plot, revealing a total of 261 differential metabolites, with 178 metabolites being upregulated and 83 metabolites being downregulated (Figure 6A). In addition, hierarchical clustering analysis of the differential metabolites showed that the rumen differential metabolites were distinct between the groups, as shown in Figure 6B. Red indicates an increase in the relative content of substances, while blue indicates a decrease in the relative content of substances. The intra-group clustering of the control group and the ZnONP group was ideal. Compared to the CK group, the expression levels of nicotinamide riboside, guanosine, and inosine were upregulated in the ZnONP group, while the expression levels of gallocatechin, pipecolic acid, and rosmaricine were downregulated (Table 4). After adding ZnONPs to the diet, significant changes occurred in the rumen metabolism of dairy goats.

**Table 4:** Information on antioxidant-related differential metabolites.

Name	Vip	*p*-value	Fold_Change	Up/down	Cas
Gallocatechin	1.6812	0.0186	0.4217	Down	970-73-0
Pipecolic acid	1.5617	0.0316	0.4362	Down	535-75-1
Rosmaricine	1.4282	0.0486	0.5960	Down	3650-11-1
Glutamine	1.7095	0.0121	1.5842	Up	56-85-9
Ribose	1.9243	0.0037	4.0090	Up	24259-59-4
Allopurinol	1.9125	0.0053	1.5999	Up	315-30-0
Anserine	1.4803	0.0299	1.9045	Up	584-85-0
Rhapontigenin	1.6332	0.0229	2.0741	Up	500-65-2
Nicotinamide riboside	1.6287	0.0232	1.5632	Up	1341-23-7
Guanosine	1.3605	0.0489	1.8402	Up	118-00-3
Inosine	2.0555	0.0031	1.7788	Up	58-63-9

Vip, variable importance in projection. Fold_Change, fold change in the metabolite concentration. Up/Down, Levels of metabolites adjusted upward/downward. Cas, Chemical Abstracts Service.

#### 3.3.3 The effects of ZnONPs on the rumen metabolic pathways of dairy goats

**Figure 7:**
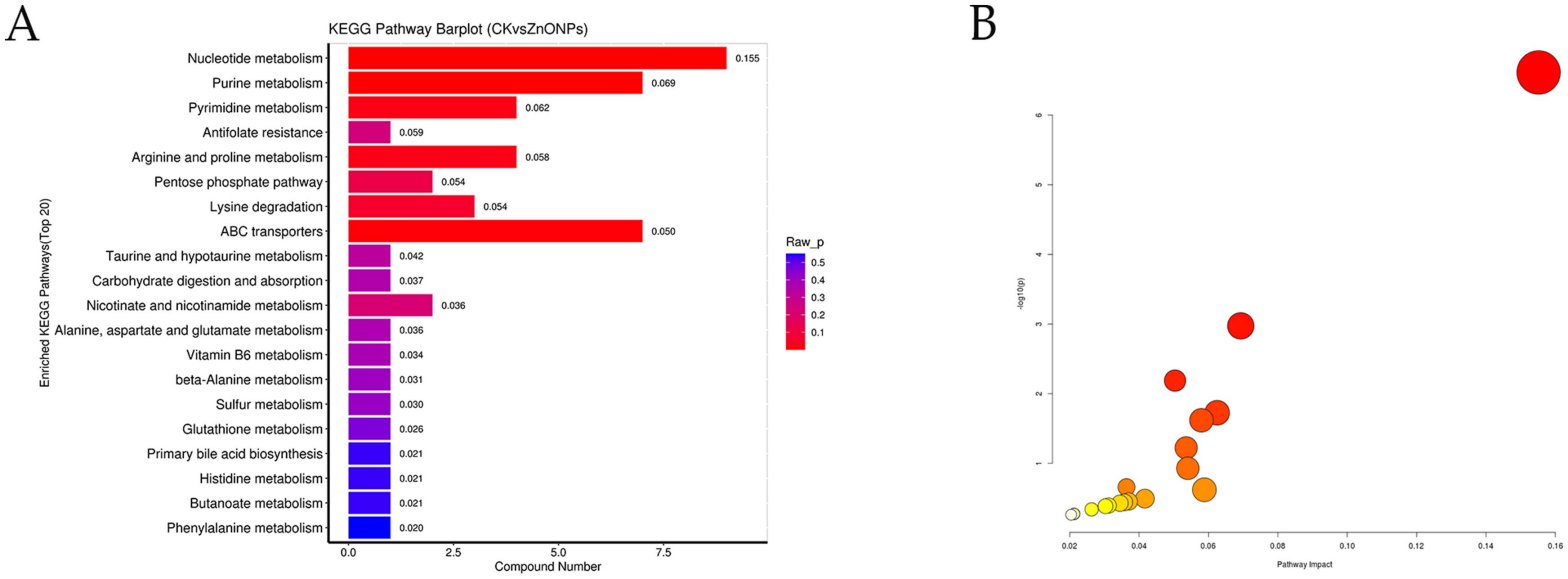
KEGG pathway enrichment bar chart **(A)**. The color represents the *p*-value of the enrichment analysis, with a deeper red color indicating a more significant degree of enrichment. Analysis of metabolic pathways in each comparative combination **(B)**. The color of the bubble represents the *p*-value of the enrichment analysis, with a deeper red color indicating a more significant degree of enrichment. The size of the dots represents the number of differential metabolites enriched in that pathway. CK, dairy goats fed a standard diet. ZnONPs, dairy goats fed a standard diet plus 30 mg ZnONPs/kg DM.

The detected differential metabolites were screened and entered into the differential pathway enrichment database, and the enriched pathways were visualized as bar graphs and bubble plots to visualize the data. Based on the significance index *p*-value, the pathways with significantly different metabolisms were selected. The top 20 pathways with the smallest *p*-values are shown in Supplementary Table S3, and the KEGG graph represents the enriched metabolic pathways, with significantly different metabolic pathways reflected in the bubble plot (Figure 7). Among them, the five pathways with the most significant changes (*p* < 0.05) were nucleotide metabolism, purine metabolism, ABC transporters, pyrimidine metabolism, and arginine and proline metabolism.

### 3.4 Correlation of metabolites and lactation with rumen microbiota

**Figure 8:**
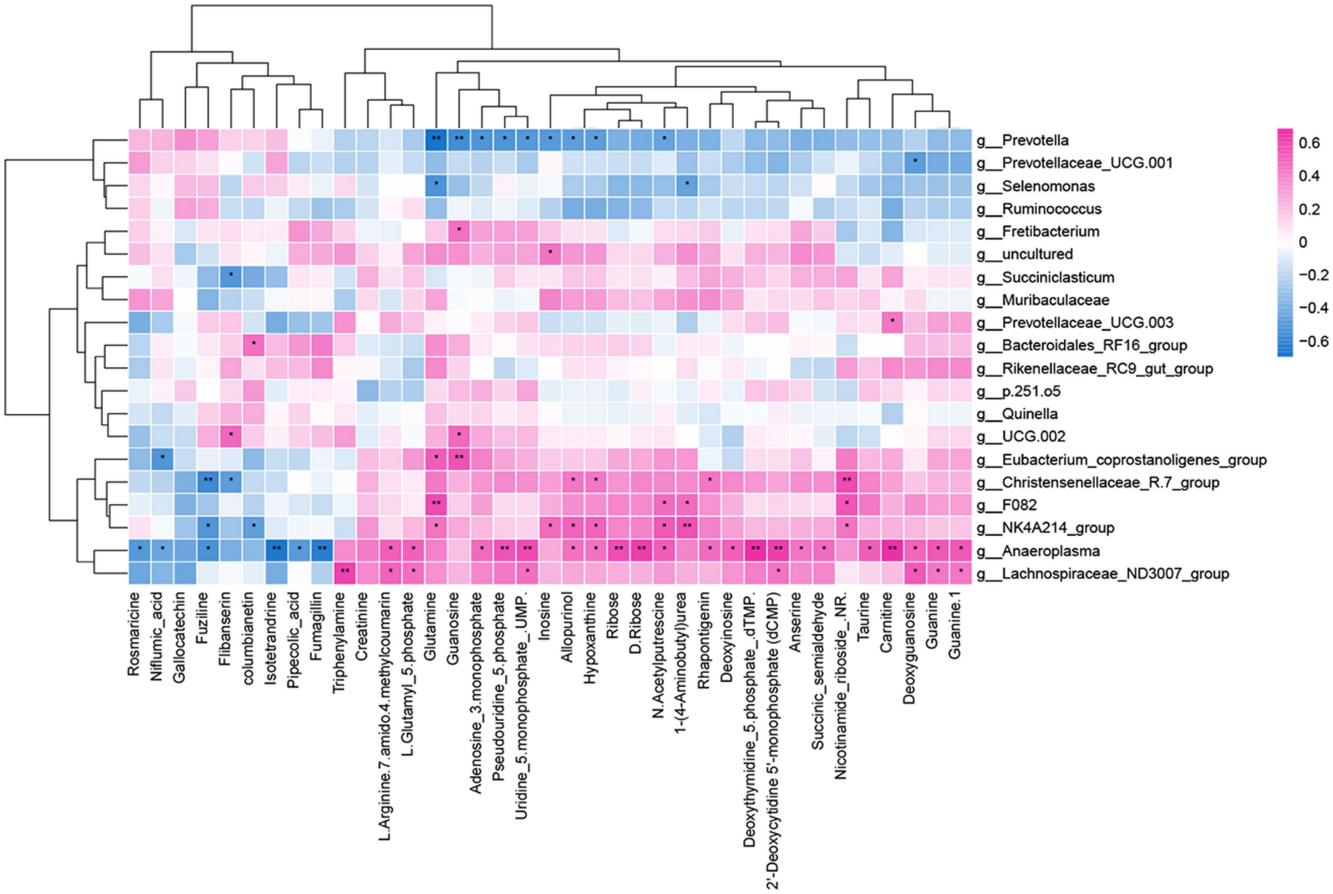
Rumen microbiome–metabolome correlation heatmap. *p < 0.05 and **p < 0.01. Red represents a positive correlation, and blue represents a negative correlation.

Through Spearman’s correlation analysis, it was found that there was a correlation between differential metabolites and differential bacterial genera. The correlation was visualized as a heatmap (Figure 8), where red squares represent positive correlations, blue squares represent negative correlations, and the intensity of color indicates the strength of the correlation coefficient, with darker colors indicating higher coefficients and lighter colors indicating lower coefficients.

In the rumen, metabolites involved in nicotinate and nicotinamide metabolism, arginine and proline metabolism, and purine metabolism were primarily focused on the significance index *p*-value for selection. Nicotinamide riboside in nicotinate and nicotinamide metabolism showed a highly significant positive correlation with *Christensenellaceae_R.7_group*, while succinic semialdehyde exhibited a significant positive correlation with *Anaeroplasma*. In arginine and proline metabolism, N-acetylputrescine showed significant positive correlations with *NK4A214_group*, *Anaeroplasma*, and *F082* and a significant negative correlation with *Prevotella*. 1-(4-Aminobutyl) urea exhibited a highly significant positive correlation with *NK4A214_group* and a significant negative correlation with *Selenomonas*. L-Glutamyl 5-phosphate demonstrated significant positive correlations with *Lachnospiraceae_ND3007_group* and *Anaeroplasma*. In purine metabolism, deoxyinosine exhibited a significant positive correlation with *Anaeroplasma*, while inosine showed significant positive correlations with *NK4A214_group* and significant negative correlations with *Prevotella*. Guanosine demonstrated a highly significant positive correlation with *Eubacterium_coprostanoligenes_group* and a highly significant negative correlation with *Prevotella*. Hypoxanthine displayed a significant positive correlation with *Christensenellaceae_R.7_group* and a significant negative correlation with *Prevotella*. Moreover, deoxyguanosine and guanine were significantly positively correlated with *Lachnospiraceae_ND3007_group* and *Anaeroplasma*, while Adenosine 3-monophosphate exhibited significant positive correlations with *Anaeroplasma* and significant negative correlations with *Prevotella*.

As shown in Table 5, *Quinella* is significantly positively correlated with milk fat and shows no significant correlation with other indicators. The beneficial bacteria *Bacteroidales_RF16_group* are significantly positively correlated with milk protein, lactose, total solids, and non-fat milk solids and show no significant correlation with milk fat. Milk yield was significantly positively correlated with beneficial bacteria (*RF39* and *Clostridia vadinBB60 group*) and significantly negatively correlated with *Succinivibrio*.

**Table 5:** Correlation between rumen microbiota and lactation.

Genus	Fat	Lactose	Protein	Total solids	Non-fat milk solids	Milk yield
*Quinella*	0.444*	0.086	0.019	0.047	0.047	0.156
*Succinivibrio*	−0.209	−0.105	−0.055	−0.049	−0.077	−0.448*
*RF39*	−0.324	−0.08	−0.048	−0.077	−0.062	0.524*
*Clostridia_vadinBB60_group*	−0.313	0.294	0.281	0.266	0.297	0.475*
*Bacteroidales_RF16_group*	0.324	0.511*	0.496*	0.501*	0.508*	−0.004

*p < 0.05.

## 4 Discussion

Zinc has a positive effect on the production performance of ruminants. Appropriate use can enhance the digestion and absorption of feed, increase feed efficiency, and, to some extent, improve intestinal health, thereby effectively increasing milk yield (Wang et al., 2013; Oconitrillo et al., 2024). In this study, dietary supplementation with ZnONPs resulted in a significant increase in milk production. This result is consistent with previous research (Abdollahi et al., 2020), indicating that the appropriate addition of ZnONPs positively impacts milk production in ruminants. This may be related to the small particle size and high chemical activity of ZnONPs. It can enhance the integrity of the intestinal mucosal barrier, improve the balance of the gut microbiota, increase the efficiency of food digestion and absorption, and increase the supply of energy and nutrients, thereby effectively improving production performance (Liu et al., 2021). In this study, dietary supplementation with ZnONPs significantly increased milk fat content, with no significant impact on milk protein and lactose content. The lack of significant effect of ZnONPs on milk protein indicates that the addition of zinc to the diet does not have a noticeable impact on rumen protein metabolism in dairy goats. However, some studies have shown that adding zinc to the feed of Holstein cows significantly reduced milk fat content, with no significant impact on milk protein and lactose content (Nayeri et al., 2014). In addition, Hosseini-Vardanjani et al. (2020) found that adding ZnONPs to the diet of ewes had no significant effect on the content of protein, fat, and lactose in milk. This discrepancy may be related to factors such as the dosage and duration of feeding. Existing research on the effects of ZnONPs on milk quality in ruminants is limited, and further studies are needed to explore its mechanisms of action on lactation in ruminants.

Based on our research findings, dietary supplementation with ZnONPs significantly impacted the structure of the rumen microbiota in dairy goats. The primary bacterial phyla in the rumen were *Bacteroidota*, *Firmicutes*, and *Synergistota*, with *Bacteroidota* being the dominant phylum, consistent with previous studies (Pitta et al., 2016; Zhang et al., 2017). In this study, dietary supplementation with ZnONPs increased the relative abundance of *Bacteroidota*. Previous research has shown that *Bacteroidota* play a crucial role in promoting the production of volatile fatty acids, thereby enhancing the host’s energy utilization efficiency (Pandit et al., 2018; Söllinger et al., 2018). Hence, the promotion of *Bacteroidota* by ZnONPs could ultimately improve animal production performance by enhancing energy metabolism efficiency. Notably, our study found that *Prevotella* was predominant in the rumen, with its relative abundance significantly higher in the ZnONP group compared to the control group, indicating its high representation in the rumen microbiota (Bekele et al., 2010; Wang et al., 2017; Petrič et al., 2021; Betancur-Murillo et al., 2023). *Prevotella* effectively degrades hemicellulose through its hemicellulase, producing short-chain fatty acids that protect the gut and benefit host health (Emerson and Weimer, 2017; Schofield et al., 2018). This finding suggests that ZnONPs might enhance the fiber digestion capacity of goats, thereby improving their production efficiency. In addition, ZnONP supplementation altered the abundance of certain important bacteria, such as increasing the abundance of the *Rikenellaceae_RC9_gut_group*, which plays a significant role in the degradation of carbohydrates and the digestion of crude fiber (Pitta et al., 2010; Guo et al., 2023). The increase in such functional microbial groups could further promote the nutritional absorption and conversion in dairy goats to some extent. ZnONPs also exhibit potential anti-inflammatory properties. Gronbach et al. (2014) found that a low abundance of *Enterobacteriaceae* and a high abundance of *Bacteroidota* effectively alleviated colitis symptoms in mice. In this study, the increase in *Bacteroidota* following ZnONP supplementation may be attributed to changes in *Prevotella* abundance. *Prevotella* can produce immunosuppressive LPS, suggesting that ZnONPs have potential anti-inflammatory properties (d'Hennezel et al., 2017; Mangalam et al., 2017; Itano et al., 2023). This characteristic is of great importance for maintaining animal health, especially in dairy goat populations under high production pressure. Notably, ZnONPs may exert an inhibitory effect on pathogenic bacteria. We observed a weakening in the pentose phosphate pathway, indicating that ZnONPs might inhibit the growth of pathogens such as *Staphylococcus aureus* via this pathway (Kim et al., 2023). This antimicrobial effect could potentially reduce disease incidence and improve animal health. In summary, ZnONPs demonstrate the potential to enhance production performance and health in dairy goats by optimizing the structure and function of the rumen microbiota.

The supplementation of ZnONPs in the diet significantly altered the metabolite composition in the rumen of dairy goats, revealing its profound impact on metabolic pathways. These changes are significant not only at the metabolic level but also for animal production and health. Yan et al. (2012) reported significant metabolic changes in the kidneys of rats following oral administration of ZnONPs (100–1,000 mg/kg) for 14 days, with elevated levels of lactate and *α*-glucose. Similarly, Lee et al. (2016) observed that acute inhalation of ZnONPs altered lung metabolism in rats. While these studies have focused on metabolic changes in the kidneys and lungs, the effects of ZnONPs on the rumen metabolism of ruminants remain unclear. Purine metabolism plays a crucial role in alleviating oxidative stress damage, which is essential for maintaining the stability of rumen tissue (Manzoni et al., 2020). Xiong et al. (2022) found that purine metabolism disorders led to increased adenine content in the feces of mice with liver fibrosis, causing oxidative stress damage to the liver. In this study, we observed an upregulation of guanosine and inosine levels following the addition of ZnONPs, which had a positive impact on purine metabolism. This reduced the risk of diseases associated with oxidative stress and contributed to improved health in dairy goats. In addition, the supplementation of ZnONPs increased the levels of L-arginine, which is significantly enriched in the arginine and proline metabolism pathways. Similarly, Zhang et al. (2018) found that after 24 weeks of oral administration of ZnONPs to hens, the level of arginine in plasma metabolites increased significantly, promoting hepatic lipid metabolism and enhancing growth performance. This suggests that the upregulation of L-arginine levels following ZnONP supplementation may contribute to improved growth and immune function in dairy goats. Studies have shown that nicotinamide riboside (NR) participates in intracellular redox reactions and energy metabolism through the activation of sirtuin 1 in macrophages stimulated by alcohol, exerting anti-inflammatory and antioxidant effects (Kourtzidis et al., 2018; Kang et al., 2021). In this study, dietary supplementation with ZnONPs exhibited an upregulation of NR levels, which may help mitigate oxidative stress-induced cellular damage and positively impact the growth efficiency of dairy goats. In summary, ZnONPs significantly enhance the immunity and antioxidant capacity of dairy goats by modulating metabolites and affecting metabolic pathways, thereby contributing to the stability of metabolic and physiological functions.

Many studies indicate a close relationship between the rumen microbiota and production performance (Li et al., 2024; Liu et al., 2024). Zhou et al. (2023) found that an increase in the relative abundance of rumen coccus can effectively improve the production performance of goats. In our study, milk yield was significantly positively correlated with probiotics such as *RF39* and *Clostridia_vadinBB60_group* and significantly negatively correlated with *Succinivibrio*. The *RF39* is generally considered to be one of the microbial groups closely associated with the rumen fermentation process (Tilahun et al., 2024). *Clostridia_vadinBB60_group* contributes positively to production performance by degrading cellulose and other complex carbohydrates to promote the production of volatile fatty acids, which provide the necessary energy for ruminants (Xiao et al., 2021). In addition to milk yield, milk fat was also closely associated with rumen microbiota. Xue et al. (2018) found that milk fat was positively correlated with the relative abundance of *Butyrivibrio, Pseudobutyrivibrio*, and *Clostridium*. In this study, we found that the relative abundance of *Quinella* was positively correlated with milk fat content. The *Quinella* produces beneficial metabolites (such as lactic acid, alcohols, and volatile fatty acids) through carbohydrate fermentation, effectively improving the host digestive metabolism (Gruninger et al., 2014). Therefore, our results corroborate the finding that production performance is strongly correlated with rumen microbiota (Li et al., 2024; Liu et al., 2024). In addition, ZnONPs enhance milk yield, and milk fat might be associated with increased *RF39* and *Clostridia_vadinBB60* and increased *Quinella*, respectively.

In this study, correlation analysis also revealed the relationships between differential metabolites and microbial taxa, further emphasizing the crucial role of the rumen microbiome in animal production. This study has found that *Anaeroplasma* was positively correlated with most metabolites including deoxyinosine, guanine, and deoxyguanosine. Importantly, these metabolites are significantly enriched in purine metabolism, which can effectively enhance antioxidant capability (Furuhashi, 2020). As a bacterium with anti-inflammatory effects, *Anaeroplasma* can enhance the integrity of the intestinal mucosal barrier by inducing the anti-inflammatory cytokine TGF-*β*, thereby increasing the level of mucosal IgA (von Goetze et al., 2022). In addition to *Anaeroplasma*, *Christensenellaceae_R.7_group*, a common genus within *Firmicutes*, is considered a beneficial bacterium (Zhang et al., 2020; Jiang et al., 2022; Yan et al., 2022). In the present study, dietary supplementation with ZnONPs increased the relative abundance of *Christensenellaceae_R.7_group* and the levels of nicotinamide riboside. The *Christensenellaceae_R.7_group* was significantly positively correlated with nicotinamide riboside, which can reduce the damage caused by oxidative stress (Hong et al., 2018). Combining previous findings with this study highlights the importance of ZnONPs in improving antioxidant and anti-inflammatory effects through the regulation of *Anaeroplasma* and *Christensenellaceae_R.7_group* and related metabolites. This further emphasizes the critical role of ZnONPs in metabolic activities that enhance animal health by regulating specific rumen microbiota.

The safety assessment of ZnONPs as a novel material is crucial. Although the European Union has explicitly banned the use of zinc oxide in piglet feed since 2022 due to excessive use of zinc oxide causing zinc overload and environmental pollution, low doses of ZnONPs have a positive effect on animal production, with no potential adverse effects observed. Compared to commercial zinc oxide, the larger surface area and higher bioavailability of ZnONPs enhance absorption efficiency within the animal digestive system, which can achieve a good effect at lower doses (25–100 mg/kg diet) (Oh et al., 2022). In addition, many studies have found that low doses of ZnONPs (20–40 mg/kg DM) contributed to maintaining the health and productive performance of sheep and ewes (Alijani et al., 2020; Hosseini-Vardanjani et al., 2020). In this study, the ZnONPs used had an average particle size of approximately 30 nm and a purity of 99.9%, which effectively enhanced the biological activity of zinc oxide and reduced the adverse effects of impurities in commercial zinc oxide. This study selected 30 mg ZnONPs/kg DM as the supplementation dose, which was considered to be safe and beneficial to dairy goats and was in line with dosages used in previous research. Although the genotoxicity of ZnONPs remains controversial, low doses of ZnONPs did not induce genotoxicity, which has been demonstrated in multiple studies (Kwon et al., 2014; Mahmoud et al., 2021; Gencyilmaz et al., 2024). Therefore, the low doses (30 mg/kg DM) of ZnONPs used in this study might not have potential genotoxic effects in dairy goats; further *in vivo* studies and long-term toxicological assessments are needed to determine the specific risks.

## 5 Conclusion

The results indicated that the addition of ZnONPs to the diet can increase milk yield and milk fat content in dairy goats and enhance the richness and diversity of the rumen microbiota. The rumen metabolism of dairy goats exhibited significant changes, resulting in the identification of 261 differential metabolites. There is a significant correlation between differential bacteria and metabolites. Milk yield is significantly positively correlated with beneficial bacteria (*RF39* and *Clostridia vadinBB60 group*), and milk fat content is significantly positively correlated with *Quinella*. ZnONPs enhance lactation performance and regulate the production of metabolic products by modulating the structure of the rumen microbiota in dairy goats.

## Data Availability

The data presented in the study are deposited in the NCBI repository, accession number PRJNA1173358.

## Footnotes

^1^ http://www.kegg.jp
